# JNK‐IN‐8 treatment improves ARDS‐induced cognitive impairment by inhibiting JNK/NF‐κB‐mediated NLRP3 inflammasome

**DOI:** 10.1002/brb3.2980

**Published:** 2023-03-29

**Authors:** Yunchao Shi, Ying Fang, Peng Shen, He Liu, Longsheng Xu, Liyan Wang, Maoxian Yang

**Affiliations:** ^1^ Department of Intensive Care Unit The First Hospital of Jiaxing & First Affiliated Hospital of Jiaxing University Jiaxing China; ^2^ Department of Pathology The First Hospital of Jiaxing & First Affiliated Hospital of Jiaxing University Jiaxing China; ^3^ Department of Anesthesiology The Affiliated Huzhou Hospital Zhejiang University School of Medicine & Huzhou Central Hospital Huzhou China; ^4^ Department of Central Laboratory The First Hospital of Jiaxing & First Affiliated Hospital of Jiaxing University Jiaxing China; ^5^ Department of General Practice The First Hospital of Jiaxing & First Affiliated Hospital of Jiaxing University Jiaxing China

**Keywords:** acute respiratory distress syndrome, cognitive impairment, JNK/NF‐κB pathway, JNK‐IN‐8, NLRP3 inflammasome

## Abstract

**Purpose:**

Cognitive impairment is a critical complication of acute respiratory distress syndrome (ARDS). However, effective interventions are lacking. Growing evidence demonstrates that c‐Jun N‐terminal kinase (JNK)‐mediated neuroinflammation is involved in the development of ARDS. Therefore, we hypothesized that the JNK pathway is involved in ARDS‐induced cognitive impairment.

**Methods:**

An in vivo rat model of ARDS was established by treating it with lipopolysaccharide. The cognitive function was assessed by behavioral tests. The levels of pro‐inflammatory cytokines, JNK and NOD‐, LRR‐, and pyrin domain‐containing protein 3 (NLRP3) were analyzed by enzyme‐linked immunosorbent assay, western blot, or immunohistochemical analysis.

**Results:**

We found that JNK inhibitor 8 (JNK‐IN‐8) alleviated cognitive impairment, neuroinflammation, and NLRP3 inflammasome activation in the ARDS rat model. Additionally, an in vivo study showed that the protective effect of JNK‐IN‐8 on cognitive impairment was blocked by nigericin, an NLRP3 activator.

**Conclusions:**

Our data suggest that JNK‐IN‐8 treatment improves ARDS‐induced cognitive impairment by inhibiting the JNK/nuclear factor‐κB‐mediated NLRP3 inflammasome.

## Graphical abstract

Our data suggest that JNK‐IN‐8 treatment improves ARDS‐induced cognitive impairment by inhibiting the JNK/NF‐?B‐mediated NLRP3 inflammasome.

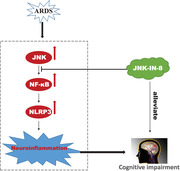

## 1 INTRODUCTION

Acute respiratory distress syndrome (ARDS) is a clinical condition associated with high fatality and sequelae rates (Force et al., 2012; Thompson et al., 2017) and is usually recognized as a complication induced by trauma, infection, and other conditions (Bellani et al., 2016). It is characterized by high pulmonary alveolar capillary permeability and intense pulmonary inflammation. Furthermore, cognitive impairment is a complication of ARDS (Huang et al., 2021). A clinical study has reported that survivors of ARDS have long‐term neurological deficits (Mart & Ware, 2020). Recently, it has been reported that patients with coronavirus disease‐19 and ARDS have increased risks of cognitive impairment and other neurological deficits (Ermis et al., 2021; Graf et al., 1990). Although great attention has been paid to ARDS in recent decades, patients with ARDS remain at a high risk of long‐term cognitive impairment, and the related mechanisms remain unclear (Brown et al., 2019; Thompson et al., 2017; Torbic & Duggal, 2018).

Previous studies have revealed that inflammation plays a critical role in the pathological course of ARDS (Bo et al., 2021; Tirunavalli et al., 2021) and cognitive impairment (Karoly et al., 2021; Kempuraj et al., 2020; Wang et al., 2020). Typical inflammatory pathways, such as the c‐Jun N‐terminal kinase (JNK)/nuclear factor (NF)‐κB (Bo et al., 2021; Xu et al., 2021; Yamamoto et al., 2021) and PI3K/Akt (Mizuta et al., 2020; Xue et al., 2021) pathways, are involved in the pathological processes of ARDS and nerve diseases. Trauma (Kempuraj et al., 2020), surgery (Wang et al., 2020), and infection (Ermis et al., 2021) can trigger inflammation, resulting in cognitive dysfunction. Interleukin (IL)‐6, an inflammatory biomarker, and neurofilament light are negatively associated with cognitive function and gray matter volume in older adults (Karoly et al., 2021). Moreover, inflammation‐associated proteins, such as tumor necrosis factor‐α (TNF‐α) (Terrando et al., 2010) and IL‐1 (Cibelli et al., 2010), trigger postoperative neuroinflammation and cognitive dysfunction by activating inflammatory molecules, such as NF‐κB and mitogen‐activated protein kinases, in the cytoplasm. In particular, NF‐κB activation leads to the transcription and functionalization of the NLRP3 inflammasome, which participates in NLRP3/caspase‐1 signaling in the pathological process of neurodegenerative diseases (Elali & Rivest, 2016; Hanslik & Ulland, 2020). Thus, inflammatory pathway regulation might be one of the strategies to improve ARDS‐induced cognitive impairment.

The JNK/NF‐κB pathway, commonly known as the typical inflammatory pathway, is involved in ARDS and neuroinflammation (Bo et al., 2021; Yamamoto et al., 2021). Moreover, JNK functions as an upstream regulator of proinflammatory cytokines in ARDS (Bo et al., 2021; Chen et al., 2020). In a previous study has shown that administration of JNK inhibitor 8 (JNK‐in‐8) alleviates lipopolysaccharide (LPS)‐induced acute lung injury by suppressing JNK/NF‐κB signaling in LPS‐induced ARDS rats (Du et al., 2021). JNK/NF‐κB pathway inhibition by azelastine reduces the production of pro‐inflammatory molecules in LPS‐stimulated BV2 microglial cells (Nguyen et al., 2021). A previous study JNK‐IN‐8 improves functional recovery by inhibiting neuroinflammation in ischemic stroke (Zheng et al., 2020). Thus, we hypothesized that JNK‐IN‐8 could improve ARDS‐induced cognitive impairment by suppressing JNK/NF‐κB signaling. We established an LPS‐induced ARDS rat model and investigated the effects of JNK‐IN‐8 on ARDS‐induced cognitive impairment and neuroinflammation.

## 2 MATERIALS AND METHODS

### 2.1 Animals

All animal procedures were performed according to the ARRIVE guidelines to minimize the number of animals used and their suffering. Male Sprague–Dawley rats (10‐week old, weighing 200–300 g) were purchased from Charles River (Beijing, China). All rats were housed in polycarbonate cages (two rats per cage) at 25 ± 2°C with humidity between 40% and 60% and a 12‐h light/dark cycle. They were provided ad libitum access to food and water. Before the end of the experiment, rats showing signs of disease or death were euthanized immediately by intraperitoneal administration of pentobarbital sodium (40 mg/kg), followed by cervical dislocation. All animal experiments were approved by the Experimental Animal Ethics Committee of Jiaxing University School of Medicine: JUMC2019‐096.

### 2.2 LPS‐induced ARDS rat model and drug administration

Rats were anesthetized by inhalation of 2% isoflurane (Sigma‐Aldrich, MO, USA) and then subjected to intratracheal instillation of LPS (5 mg/kg) from *Escherichia coli* O111:B4 (Sigma‐Aldrich) to establish an experimental ARDS model, as previously described (Liu et al., 2021; Rocha et al., 2021). Sham rats were subjected to intratracheal instillation of phosphate‐buffered saline (PBS, 100 μL).

To investigate the role of the JNK pathway in ARDS‐induced cognitive impairment, 36 rats were randomly assigned to 3 groups as follows: the control (*n* = 12), ARDS (*n* = 12), and ARDS plus JNK‐IN‐8 (*n* = 12) groups. At 24 h after ARDS induction, the ARDS plus JNK‐IN‐8 group was intraperitoneally injected with JNK‐IN‐8 (Glpbio, CA, USA; 20 mg/kg in 20% DMSO), whereas the other groups received vehicle.

To investigate the role of NLRP3 inflammasome in cognitive impairment, 24 rats were randomized into the control (*n* = 6), ARDS (*n* = 6), ARDS+JNK‐IN‐8 (*n* = 6), and ARDS+JNK‐IN‐8 plus nigericin (*n* = 6). Rats from each group were anesthetized using ketamine (100 mg/kg) and xylazine (10 mg/kg) and then fixed on a stereotaxic frame (Stoelting, IL, USA) before intracerebroventricular (ICV) injection. The injection site was set at 0.8 mm posterior to the bregma, 1.5 mm right lateral to the sagittal suture, and 3 mm below the skull. Furthermore, 10 μL of nigericin solution (8 μg/μL; GC15811; Glpbio, CA, USA) was administered to the JNK‐IN‐8 plus nigericin group through ICV injection at a rate of 0.4 μL/min with a 10 μL microsyringe (Shanghai Gaoge, Shanghai, China). Other groups were administered the same volume of vehicle (10% DMSO and 90% saline). The needle was slowly removed 7 min after injection to reduce the backflow. After suturing, the JNK‐IN‐8 plus nigericin and JNK‐IN‐8 groups received JNK‐IN‐8, and ARDS was induced in the JNK‐IN‐8, JNK‐IN‐8 plus nigericin, and vehicle groups, as indicated.

### 2.3 Behavioral tests

The Morris water maze (MWM) test was used to assess the cognitive impairment level of each group. The rats were forced to search for the platform (*d* = 18 cm; 2 cm below the water level) in a rounded water tank (*d* = 188 cm). The tank was filled with opaque water at a temperature of 25 ± 2°C. For the first week, the rats were trained to search for the platform four times a day. The rats were randomly released into one quadrant of the water. The rats were permitted to rest for 20 s upon reaching the platform. If none of the rats reached the platform within 90 s, they were placed on it for 20 s. After the last training session on the seventh day, the rats were subjected to drug or vehicle administration, as described earlier. At 24 h after LPS administration, rats in each group underwent the hidden platform test for 5 days. The platform was moved to the diagonal quadrant and other procedures were performed as indicated. After the hidden platform test, a space exploration test was conducted by removing the platform from the water tank. A rat was released in a random quadrant, and each space exploration trial lasted 90 s. EthoVision XT (Noldus, Wageningen, the Netherlands) software was used to record the escape time and swimming path during the experiment.

### 2.4 Immunohistochemistry (IHC) and immunofluorescence

At 24 h after ARDS induction, three rats from each group were deeply anesthetized with pentobarbital sodium (40 mg/kg), followed by transcardial perfusion with 100 mL PBS and 300 mL 4% paraformaldehyde (PFA; C104190; Aladdin, Shanghai, China). The hippocampal and cortex tissues were fixed in 4% PFA for 12 h and embedded in optimal cutting temperature compound (Sakura, CA, USA). Hippocampal tissue slices (50 μm) were renatured in citrate buffer (pH 6.0) at 108°C for 5 min. For immunohistochemistry (IHC), the renatured slices were pretreated with 1% H_2_O_2_ at room temperature (RT) for 15 min. After three PBS washes, the slices were masked with 10% NGS for 1 h in PBS. Slices were incubated with anti‐NLRP3 (SAB5700723; Sigma‐Aldrich) at 4°C for 24 h, followed by incubation with biotin‐labeled goat antibody against rabbit immunoglobulin G (IgG; 1:250; A0277; Beyotime) for 2 h and avidin–biotin complex for 1 h at RT. After three PBS washes, the slices were immersed in 3,3′‐diaminobenzidine solution at 37°C for 10 min.

For immunofluorescence, after masking with NGS, the slices were probed with an antibody against Iba‐1 (1:1000; PA5‐27436; Invitrogen, CA, USA) at 4°C for 24 h, followed by incubation with Alexa 488‐conjugated secondary antibody against rabbit IgG (1:250; A0428; Beyotime). Tissue slices were stained with DAPI before sealing.

### 2.5 Western blot

Three rats from each group were euthanized, as previously described, and their hippocampal tissues were collected. The tissue was homogenized in radioimmunoprecipitation assay solution (P0013C; Beyotime) containing phosphatase inhibitor (ab201112; Abcam). The membrane was probed with primary antibodies against p‐P65 (1:1000; #3033, Cell Signaling, USA), P65 (1:1000; #69994, Cell Signaling, USA), p‐JNK (1:800; sc‐293136; Santa Cruz, CA, USA), JNK (1:1000; PA5‐116884; Invitrogen, CA, USA), NLRP3 (1:3000; SAB5700723; Sigma‐Aldrich, MO, USA), anti‐ASC (1:500; AB3607; Sigma‐Aldrich), caspase‐1 (1:1500; SAB5700660; Sigma‐Aldrich), and anti‐GAPDH (1:5000; ab8245; Abcam) at 4°C for 16 h. After washing with TBST, the membranes were incubated with secondary antibodies (1:1000; A0216; Beyotime) and rabbit IgG (1:1000; A0208; Beyotime) at 37°C for 45 min. Immunoreactive blots were visualized using enhanced chemiluminescence (P0018FS; Beyotime). GAPDH was used to normalize the protein levels in each sample.

### 2.6 Enzyme‐linked immunosorbent assay (ELISA)

Commercial enzyme‐linked immunosorbent assay (ELISA) kits were used to measure TNF‐α (ml002859; mlbio, Shanghai, China), IL‐6 (ml064292; mlbio), and IL‐1β (ml102828; mlbio) levels in the hippocampal tissues of ARDS rats in accordance with the manufacturer's protocol. Hippocampal protein samples were prepared as described previously. The OD 450 nm was measured using a microplate reader (51119600DPC; Thermo Fisher Scientific).

### 2.7 Statistical analysis

All digital image data were quantified using the ImageJ software. Western blotting and ELISA were performed in triplicates. All values are displayed as the mean ± standard error of the mean. The differences between two groups were verified via Student's *t*‐test or one‐way ANOVA using SPSS 20.0. Statistical significance was set at *p* < .05.

## 3 RESULTS

### 3.1 JNK‐IN‐8 improved ARDS‐induced cognitive impairment

**FIGURE 1:**
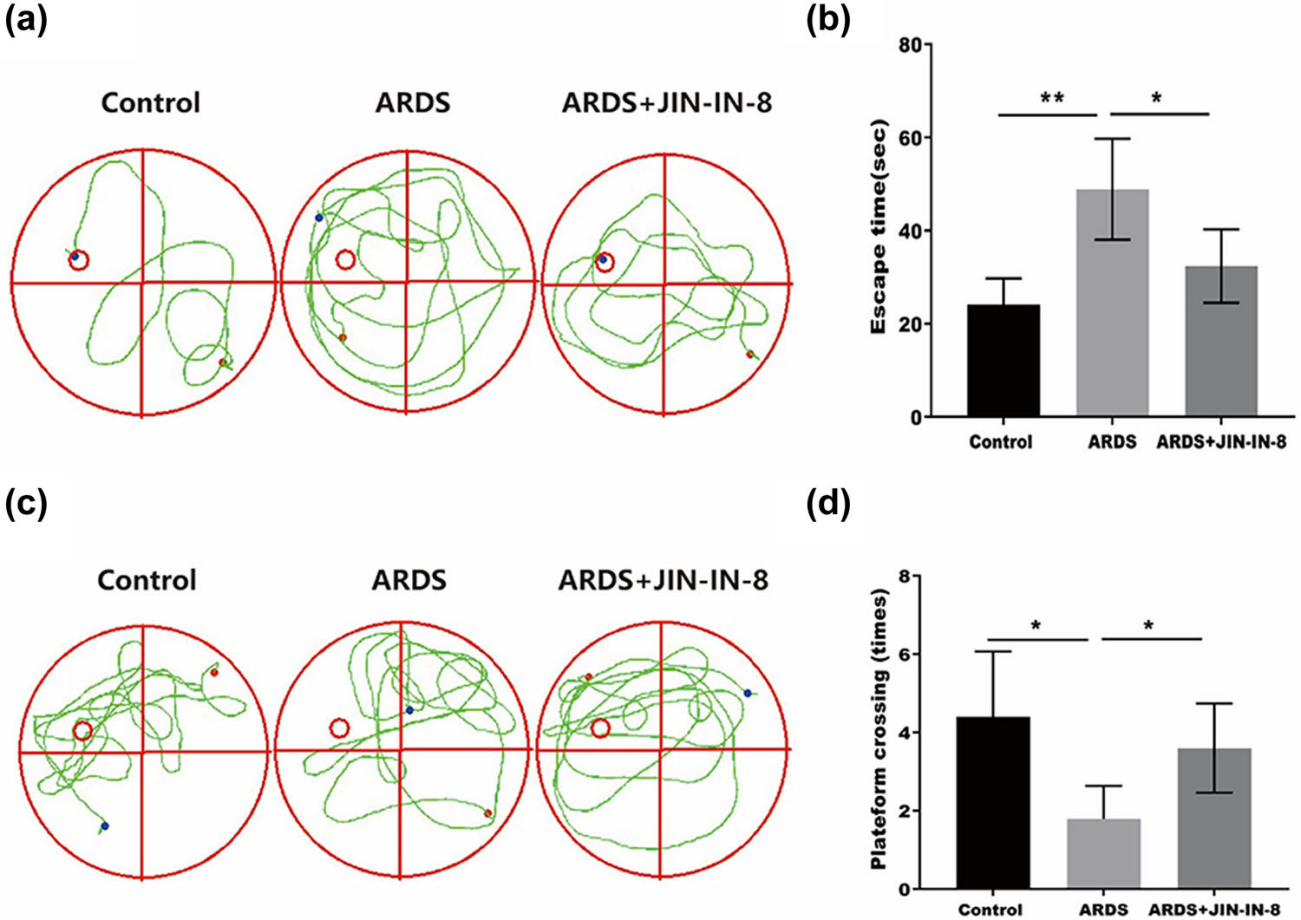
c‐Jun N‐terminal kinase inhibitor 8 (JNK‐IN‐8) improved acute respiratory distress syndrome (ARDS)‐induced cognitive impairment: (A) effects of JNK‐IN‐8 on ARDS‐induced cognitive impairment were investigated by the Morris water maze in ARDS rat model (*n* = 6); (A and B) hidden platform test; (C and D) space exploration test. *p < .05, **p < .01, and ***p < .001.

To examine the effect of JNK‐IN‐8 on ARDS‐induced cognitive impairment, we performed the MWM test. In particular, we evaluated the cognitive function of ARDS rats with or without JNK‐IN‐8 treatment. In the hidden platform test (Figure 1a), the escape time of ARDS rats was significantly longer than that of control rats. However, JNK‐IN‐8 obviously shortened the escape time of ARDS rats (Figure 1b), suggesting that ARDS affected cognitive impairment in rats and that JNK‐IN‐8 improved ARDS‐induced cognitive impairment. Furthermore, in the space exploration test (Figure 1c), the platform crossing times of ARDS rats were significantly reduced compared with those of the control rats. However, JNK‐IN‐8 obviously increased the platform crossing times of ARDS rats (Figure 1d). Our results indicate that JNK‐IN‐8 improves ARDS‐induced cognitive impairment.

### 3.2 JNK‐IN‐8 suppressed microglia‐mediated neuroinflammation after ARDS induction

**FIGURE 2:**
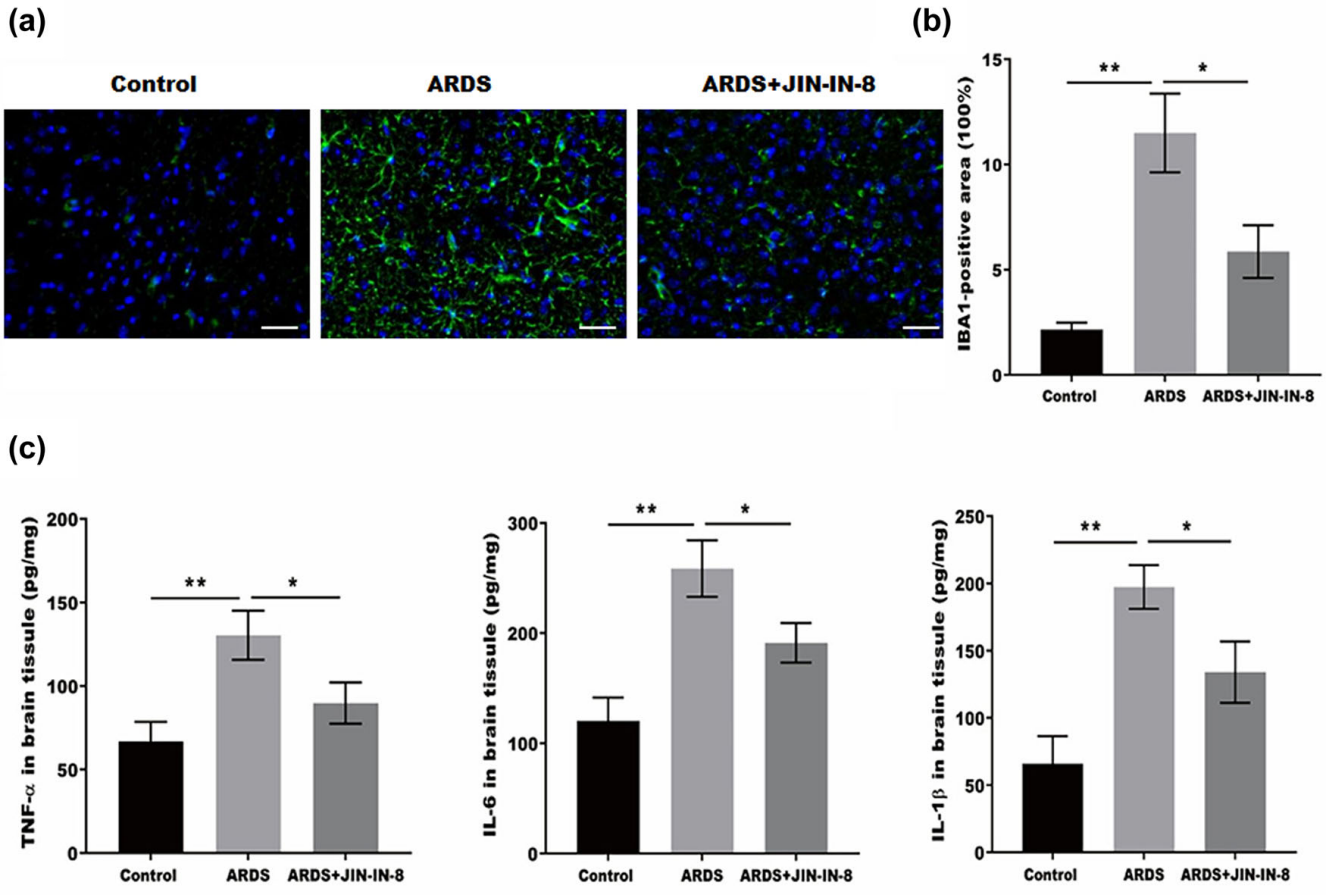
c‐Jun N‐terminal kinase inhibitor 8 (JNK‐IN‐8) inhibited microglial activation and neuroinflammation in vivo following acute respiratory distress syndrome (ARDS) induction: (A and B) microglial activation in the brain tissue of ARDS rats was analyzed by immunofluorescence. Scale bar = 50 μm; (C) tumor necrosis factor‐α (TNF‐α), interleukin (IL)‐6, and IL‐1β expression levels in the brain tissue of ARDS rats were detected by enzyme‐linked immunosorbent assay (ELISA). *p < .05, **p < .01, and ***p < .001.

We assessed microglial activation using an immunofluorescence assay to explore the underlying mechanism by which JNK‐IN‐8 improved ARDS‐induced cognitive impairment. Figure 2a,b shows that the microglial activation was markedly increased in ARDS rats, as evidenced by intensive Iba‐1‐positive staining, whereas the effect was significantly suppressed by JNK‐IN‐8.

Given that neuroinflammation plays an important role in the development of cognitive impairment, we used ELISA to assess the production of pro‐inflammatory cytokines in the brain tissues. As shown in Figure 2c, the IL‐1β, IL‐6, and TNF‐α expressions were markedly upregulated in the ARDS group, and this effect was markedly blocked by JNK‐IN‐8 treatment. This suggested that JNK‐IN‐8 suppressed microglia‐induced neuroinflammation in vivo following ARDS induction.

### 3.3 JNK‐IN‐8 suppressed JNK and NF‐κB pathway activation

**FIGURE 3:**
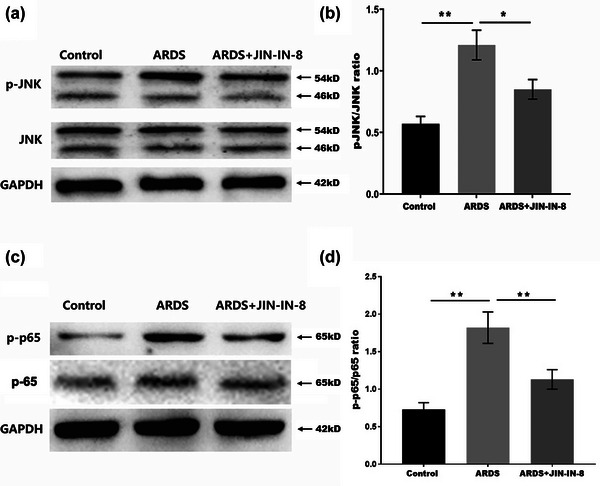
c‐Jun N‐terminal kinase inhibitor 8 (JNK‐IN‐8) suppressed JNK and nuclear factor (NF)‐κB pathway activation: (A and B) western blotting and semiquantitative analysis for phosphorylated (p)‐JNK and JNK in the brain tissue of acute respiratory distress syndrome (ARDS) rats; (C and D) western blotting and semiquantitative analysis for p‐p65 and p65 in the brain tissue of ARDS rats. GAPDH, glyceraldehyde 3‐phosphate dehydrogenase. *p < .05, **p < .01, and ***p < .001.

We assessed JNK pathway activation by western blotting. Figure 3a,b shows that p‐JNK levels in the brain tissue of ARDS rats were significantly increased compared with those of control rats, and this effect was blocked by JNK‐IN‐8.

A previous study has suggested that JNK‐IN‐8 can inhibit NF‐κB activation in LPS‐induced acute lung injury (Du et al., 2021). NF‐κB is a key upstream inducer of pro‐inflammatory cytokines in microglia. As a result, we looked into whether inhibiting JNK‐IN‐8‐induced neuroinflammation in ARDS rats is connected to the activation of NF‐κB pathway. As shown in Figure 3c,d, p‐p65 levels in ARDS rats were significantly increased compared with those in control rats, and this effect was blocked by JNK‐IN‐8.

### 3.4 JNK‐IN‐8 inhibited NLRP3 inflammasome activation

**FIGURE 4:**
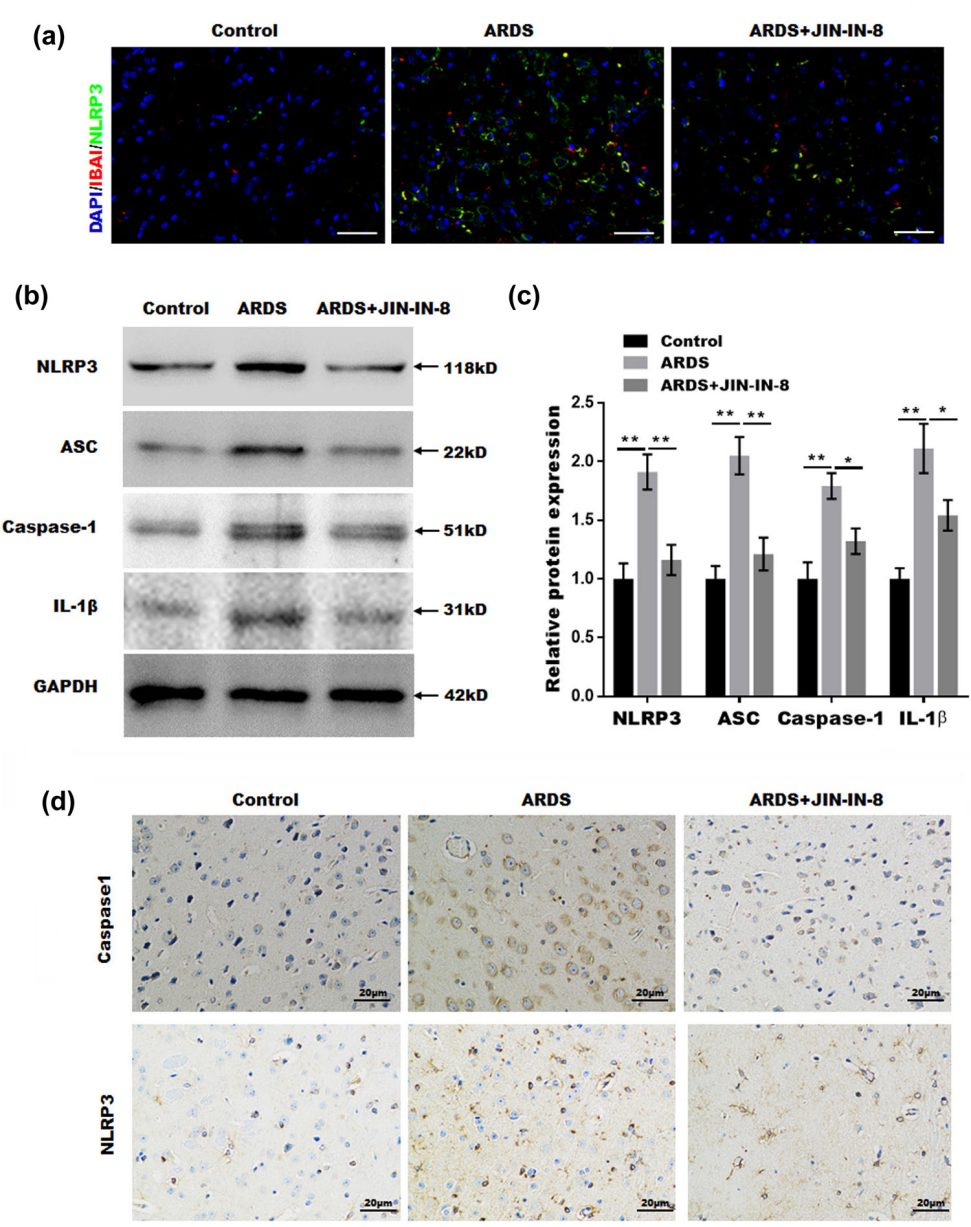
c‐Jun N‐terminal kinase inhibitor 8 (JNK‐IN‐8 inhibited NOD‐, LRR‐, and pyrin domain–containing protein 3 (NLRP3) inflammasome activation: (A) the co‐localization of NLRP3 and IBA1 in macrophages was determined by immunofluorescence. Scale bar = 50 μm; (B‐C) western blotting and semiquantitative analysis for NLRP3, apoptosis‐associated speck‐like protein containing a caspase recruitment domain (ASC), and caspase‐1 in the brain tissue; (D) NLRP3 and caspase‐1 expression in the brain tissue was detected by immunohistochemistry. Scale bar = 20 μm. GAPDH, glyceraldehyde 3‐phosphate dehydrogenase. **p < .01 and ***p < .001.

Many studies have suggested that NF‐κB plays an important role in NLRP3 activation, and that the NLRP3 activation in macrophage is considered a key mediator of neuroinflammation. Consistent with these conclusions, our results found that the activation of NLRP3 inflammasome was mainly localized in macrophages in ARDS mice model (Figure 4a). Next, we evaluated NLRP3 inflammasome activation in the brain tissue of ARDS rats. As shown in Figure 4b,c, NLRP3, ASC, caspase‐1, and IL‐1β expressions were markedly increased in ARDS rats, and this effect was blocked by JNK‐IN‐8. More importantly, in addition, IHC staining for NLRP3 and caspase‐1 in the cortex of brain tissue further confirmed this conclusion (Figure 4d). Therefore, our results indicate that JNK‐IN‐8 treatment inhibited NLRP3 inflammasome activation in ARDS rat brain tissue.

### 3.5 JNK‐IN‐8 improved ARDS‐induced cognitive impairment by mediating NLRP3 inflammasome activation

**FIGURE 5:**
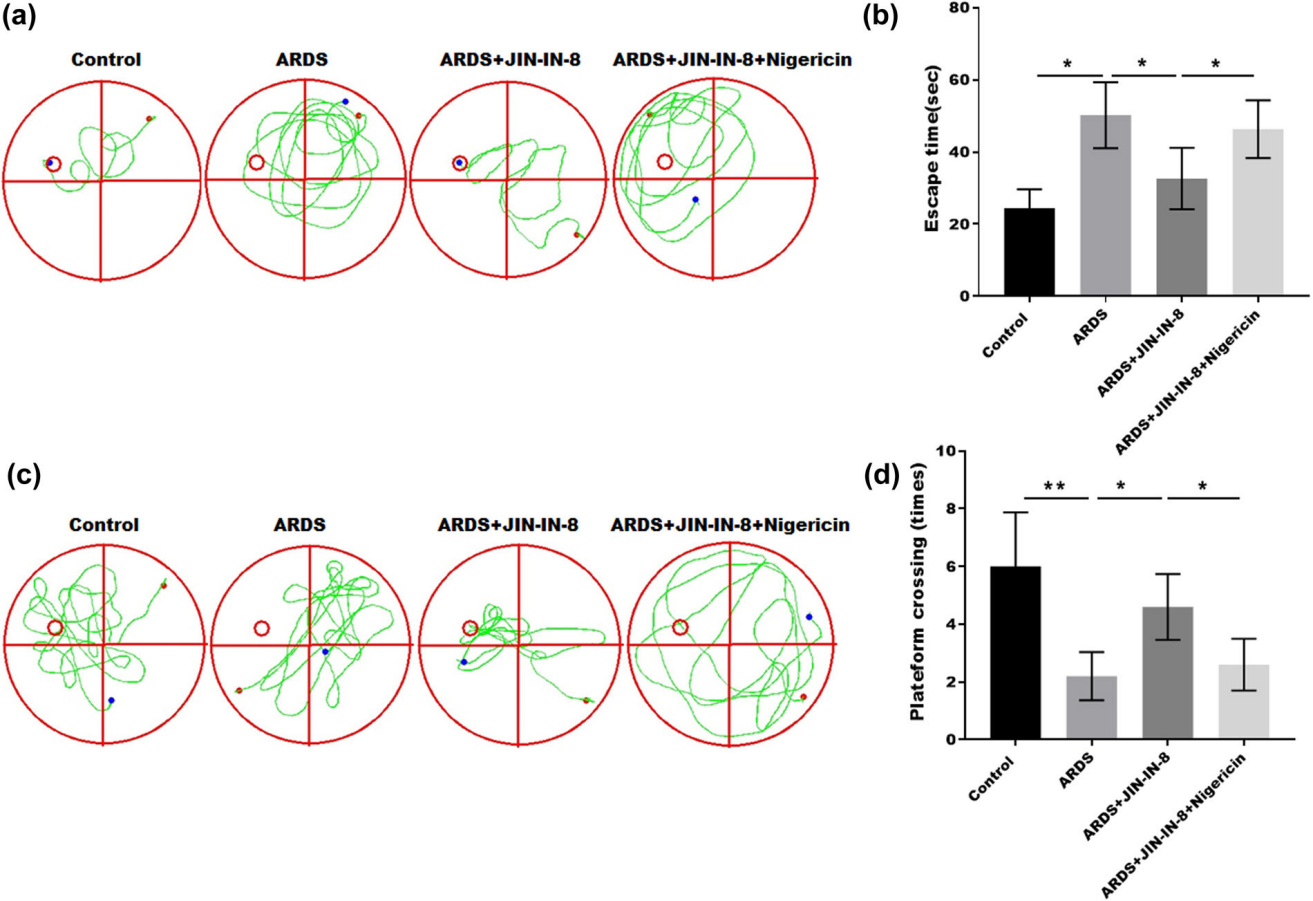
c‐Jun N‐terminal kinase (JNK) inhibitor 8 (JNK‐IN‐8) improved acute respiratory distress syndrome (ARDS)‐induced cognitive impairment by activating the NLRP3 inflammasome. Cognitive function of ARDS rats treated with JNK‐IN‐8 with or without nigericin: (A and B) hidden platform test; (C and D) space exploration test. *p < .05, **p < .01, and ***p < .001.

To confirm that the treatment effects of JNK‐IN‐8 are dependent on NLRP3 activation, we used nigericin, an NLRP3 activator, to regulate NLRP3‐mediated inflammation. We performed the MWM test to investigate the cognitive function of ARDS rats treated with JNK‐IN‐8 with or without nigericin. Figure 5a,b shows that in the hidden platform test, the escape time prolonged by ARDS was markedly decreased by JNK‐IN‐8 treatment; however, this effect was reversed by nigericin treatment. Consistently, in the space exploration experiment, the platform crossing times decreased by ARDS were markedly increased by JNK‐IN‐8 treatment; however, this effect was reversed by nigericin treatment (Figure 5c,d). Together, these data suggest that JNK‐IN‐8 improves ARDS‐induced cognitive impairment by mediating NLRP3 inflammasome activation.

## 4 DISCUSSION

ARDS is a common and serious acute lung injury, with high morbidity and mortality rates. Furthermore, cognitive impairment is a critical complication of ARDS. A previous study has suggested that JNK‐mediated neuroinflammation is involved in ARDS‐induced cognitive impairment. However, the underlying mechanism remains unclear. In the current study, we demonstrated that JNK‐IN‐8 treatment improved ARDS‐induced cognitive impairment by JNK/NF‐κB‐mediated NLRP3 inflammasome suppression, as evidenced by the following results: (Force et al., 2012) JNK‐IN‐8 improved ARDS‐induced cognitive impairment; (Thompson et al., 2017) JNK‐IN‐8 suppressed microglia‐mediated neuroinflammation after ARDS induction; (Bellani et al., 2016) JNK‐IN‐8 suppressed JNK and NF‐κB pathway activation; (Huang et al., 2021) JNK‐IN‐8 inhibited NLRP3 inflammasome activation; (Mart & Ware, 2020) JNK‐IN‐8 improved ARDS‐induced cognitive impairment by regulating the activation of NLRP3 inflammasome. Our results indicate the potential of JIN‐IN‐8 in the treatment of ARDS‐induced cognitive impairment.

Previous research has revealed that ARDS‐induced cognitive dysfunction may be caused by a variety of factors. One of these factors is hypoxemia, which is often seen in ARDS patients due to impaired alveolar gas exchange and mechanical ventilation. Prolonged hypoxemia can lead to cerebral hypoxia and ultimately result in cognitive impairment. Additionally, the inflammatory response associated with ARDS, which can spread throughout the body, including the brain, may also contribute to cognitive dysfunction by damaging brain cells. Another factor is hypotension, which is frequently experienced by ARDS patients and can lead to inadequate cerebral blood flow, causing hypoxia and nutrient deprivation that can further impact cognitive function. Furthermore, medications used to treat ARDS, such as sedatives and muscle relaxants, may also affect the brain and contribute to cognitive impairment (Sasannejad et al., 2019). Among these factors, neuroinflammation has been identified as a significant pathogenic mechanism of ARDS‐induced cognitive impairment. However, despite the recognition of this mechanism, appropriate interventions to suppress neuroinflammation remain lacking. JNK signaling is associated with microglial activation, neuroinflammation, and the development of cognitive impairment. In addition, NF‐κB signaling, a key upstream inducer of pro‐inflammatory cytokines (Cheng et al., 2022), is activated by JNK. Therefore, several JNK inhibitors have been used to improve cognitive function. Carboni et al. (2008) suggested that AS601245 suppresses the activation of JNK and improves long‐term memory by inhibiting neurodegeneration. Sharma et al. (2010) demonstrated that SP600125 treatment ameliorates neurodegenerative disorders associated with oxidative stress and cognitive impairment. In the current study, we found that JNK‐IN‐8 significantly improved ARDS‐induced cognitive impairment. Microglial activation and IL‐1β, IL‐6, and TNF‐α expressions were upregulated by ARDS, whereas this effect was blocked by JNK‐IN‐8 treatment. These results suggest that JNK‐IN‐8 treatment might improve cognitive impairment by suppressing microglial activation and pro‐inflammatory responses. In addition, we verified that JNK‐IN‐8 weakened the NF‐κB signaling activation.

A growing number of studies have shown that NLRP3 inflammasomes are regulated by NF‐κB signaling (Wang et al., 2018; Yu et al., 2017). Furthermore, the NLRP3 inflammasomes contribute to the microglia‐mediated neuroinflammation (Heneka et al., 2014). Therefore, we hypothesized that JNK‐IN‐8 inhibits neuroinflammation by regulating NLRP3 inflammasome activation. As expected, our data showed that the NLRP3 inflammasome was activated in the ARDS rat brain, and that this effect was blocked by JNK‐IN‐8 treatment. More importantly, the MWM study showed that the protective effect of JNK‐IN‐8 on cognitive impairment was blocked by the NLRP3 activator nigericin, suggesting that JNK‐IN‐8 treatment improved ARDS‐induced cognitive impairment by suppressing the JNK/NF‐κB‐mediated NLRP3 inflammasome.

## CONFLICT OF INTEREST STATEMENT

The authors declare no conflict of interests.

### PEER REVIEW

The peer review history for this article is available at https://publons.com/publon/10.1002/brb3.2980.

## Data Availability

The data that support the findings of this study are available from the corresponding author upon reasonable request.
